# Vulnerable road user fatalities are associated with greater trauma: Long-term autopsy-based trends in Czechia (1969–2024)

**DOI:** 10.1371/journal.pone.0358958

**Published:** 2026-09-25

**Authors:** Michal Bíl, Richard Andrášik, Václav Svrchokryl, Martin Dobiáš, Martina Bílová

**Affiliations:** 1 CDV – Transport Research Centre, Brno, Czechia; 2 Palacky University in Olomouc, Olomouc, Czechia; Tsinghua University, CHINA

## Abstract

Traffic accidents are an inherent consequence of modern transportation and mobility systems. Changes in the post‑crash care system over the past fifty years may have influenced the survival prospects of injured vulnerable road users (VRUs) involved in traffic accidents. We therefore focused on assessing injury severity among fatally injured VRUs in Czechia. We analysed Injury Severity Scores (ISS) derived retrospectively from autopsy documentation (medical autopsy reports and forensic expert opinions) for a random sample of 15 deceased pedestrians and cyclists from each selected year (every fifth year) between 1969 and 2024 who underwent autopsy at Olomouc University Hospital (Czechia). We focused on trends in the first quartile of ISS values and applied a non‑parametric trend analysis to determine statistical significance. There was a statistically significant upward trend in the first quartile of ISS values over the study period, indicating an upward shift in the lower tail of trauma severity among fatal cases. One possible interpretation is that less severely injured VRUs may have become less represented among fatalities over time; however, this pattern cannot be attributed directly to improvements in post-crash care. It may also reflect changes in crash severity, exposure patterns, case mix, referral pathways, reporting practices, or documentation. Accordingly, Q25 ISS should be interpreted only as an indirect, hypothesis-generating proxy compatible with long-term changes in survivability, not as direct evidence that post-crash care improved.

## Introduction

Traffic accidents are an inherent consequence of modern transportation and mobility systems. They disproportionately affect younger individuals and represent avoidable failures of a traffic system that is meant to protect the lives and health of all road users [1]. According to the World Health Organization, over 1.3 million people die every year due to road traffic injuries, and between 20 and 50 million more suffer non-fatal injuries, many of which lead to long-term disabilities [2]. Vulnerable road users (VRUs)—such as cyclists and pedestrians—account for more than half of all road traffic deaths globally, particularly in urban environments.

The consequences of a traffic crash, especially in terms of injury severity, are shaped by the dynamics of the accident process, which is commonly divided into three key phases: the pre-crash phase (conditions leading to the crash), the crash phase (the moment of impact), and the post-crash phase (emergency response and medical care) [3,4]. While motor vehicle occupants have increasingly benefited from both active and passive vehicle safety improvements, VRUs remain physically unprotected during the crash phase and are therefore far more vulnerable to severe or fatal outcomes. As they are directly exposed to impact forces, even low-speed collisions can result in life-threatening trauma [5].

Unlike the crash phase, in which VRUs lack physical protection, the post-crash phase offers the potential to mitigate the consequences of injury through efficient emergency response and trauma care. Over the past decades, the development of post-crash care has contributed significantly to improved survival rates and outcomes in road traffic injury cases, particularly in high-income countries. This includes improvements in EMS response times, prehospital triage and stabilisation, on-scene medical care, and rapid transportation to specialised trauma centres ([6]).

Legislation, training, and public education have also played vital roles in strengthening emergency systems. Countries such as Sweden and the Netherlands have developed coordinated rescue chains with high standards for first aid and medical communication protocols [1,7]. International evidence clearly shows that systemic improvements in post-crash care contribute to a substantial reduction in both short- and long-term consequences of road injuries.

Evaluations of organised trauma systems and emergency medical services (EMS) consistently report reductions in preventable mortality and improved outcomes after severe injury, especially where rapid triage, timely transport, and definitive trauma care are well integrated. Importantly, these improvements can also change the *composition* of fatal cases over time: as post-crash care improves, a growing share of patients with moderate-to-severe trauma survive, and the remaining fatalities increasingly concentrate among the most critically injured individuals (a survivorship/composition shift). In such a setting, summary measures of injury severity among fatalities—particularly the lower tail of the severity distribution—may increase even if the underlying crash exposure is stable, because deaths progressively represent a more selected subset of extreme injuries.

### Injury-severity measurement and ISS ceiling effects

A practical challenge in long-term fatal-injury series is that the Injury Severity Score (ISS) is bounded above (maximum 75) and can be effectively *top-coded* in datasets dominated by very severe trauma (e.g., when any AIS equals 6). In such circumstances, central tendency measures (mean/median) can be uninformative because many observations accumulate at the ceiling. Prior work dealing with ISS saturation commonly relies on distributional summaries (e.g., quantiles), categorical cut-points (e.g., ISS ≥ 16/25 or ISS = 75), alternative severity formulations (e.g., NISS), or statistical approaches tailored to bounded/top-coded outcomes. In this study, focusing on the first quartile (Q25) provides a stable indicator of severity changes in the *lower* part of the fatality distribution that is less distorted by the ISS ceiling while remaining interpretable within the established trauma-scoring framework.

### Central and Eastern Europe context and evidence gap

While the trauma-systems literature is extensive in North America and Western Europe, long-run, population-based evidence from Central and Eastern Europe remains comparatively sparse and often fragmented across EMS reform descriptions, hospital-based case series, and road-safety reporting — which limits empirical insight into whether the expected survivorship/composition shifts observed in mature trauma systems are also detectable in CEE settings and among VRUs in particular. This provides the contextual motivation for examining a Czech archive-based case series spanning five decades of forensic autopsy documentation. Our findings add one long-term data point from two Czech regions, but they should not be interpreted as representative of Central and Eastern Europe as a whole.

### Post-crash care development in Czechia

In Czechia, the development of emergency medical services (EMS) has undergone substantial transformation since the post-World War II period. Initially, emergency care was mostly handled by transport services or general practitioners through home visits. It was not until the 1970s that more centralised EMS structures emerged, primarily in larger urban areas. During this time, new monitoring technologies were introduced that improved the quality of prehospital care.

The first air rescue station was established in Prague in 1987, along with the introduction of the rendezvous system (RVS), in which a physician in a rapid-response vehicle is dispatched to assess and treat patients at the scene. Based on clinical assessment, the patient may either be transported or released, enabling optimal resource allocation. In the early 1990s, the separation between prehospital and in-hospital emergency care led to the introduction of specialised emergency departments.

Decree No. 434/1992 set a legal maximum EMS response time of 15 minutes, which led to improved coverage and system planning. The EMS system was further regionalised in 2003, and since 2014, emergency medicine has been recognised as a distinct medical specialty, improving training and the professionalisation of the field.

Despite these advances, little is known about how the evolution of the EMS system has impacted real-world outcomes, especially among vulnerable road users. In Central and Eastern Europe, empirical evidence on long-term trends in trauma outcomes remains limited.

This study aims to examine the long-term changes in the severity of injuries sustained by cyclists and pedestrians who died as a result of road traffic accidents in two Czech regions over the past 50 years. By focusing on VRUs—who are not protected by in-vehicle safety systems—this study avoids confounding effects of technological safety improvements in vehicles. Any observed changes in injury severity may therefore be interpreted as an indirect indicator of long-term changes in the post-crash phase, under explicit assumptions and with the recognition that broader changes in crash circumstances and exposure may also influence fatal injury profiles. Conceptually, if post-crash survival improves over time, the *lower tail* of the injury-severity distribution among those who still die should shift upward because individuals with less critical injuries increasingly survive (a fatality composition shift). Against this background, our contribution is not only the application of a non-parametric trend test, but (i) the use of a uniquely long autopsy-based VRU fatality series from Czechia, (ii) an explicit focus on Q25 as a severity indicator that is informative under ISS ceiling effects, and (iii) an interpretation anchored in trauma-system survivability logic for a Czech archive-based setting situated within a Central European context where long-term outcome evidence remains limited.

## Methods

### Database of deceased at Olomouc hospital

The analysed data, which were obtained during March 2025, were sourced from autopsy reports (medical autopsies) and expert opinions (forensic autopsies) archived in paper form at the Department of Forensic Medicine and Medical Law, University Hospital and Faculty of Medicine, Palacký University Olomouc. Throughout the manuscript, we refer to these sources collectively as “autopsy documentation”; this documentation covered both on-scene and in-hospital deaths, depending on the circumstances and legal pathway of the case. Medical autopsy documentation included all necessary primary data and records for subsequent ISS determination, such as EMS dispatch records, death examination forms, autopsy findings, and laboratory test results (and in in-hospital deaths, also the autopsy accompanying form). Forensic expert opinions additionally included the official court order for autopsy, outlining crash circumstances, forensic conclusions, and the relation of death to the traffic accident.

Importantly, this definition should not be interpreted as implying full population coverage of all VRU fatalities in the two regions. The archive-based sampling frame may itself have changed over the 50-year period because of temporal variation in autopsy practices, referral criteria, institutional coverage, administrative procedures, or routing of cases between facilities. Thus, the study population is best described as autopsied VRU fatalities recorded in this institutional archive, not all VRU fatalities occurring in the regions.

Across the study period, autopsies in cases of sudden, violent, or suspicious death have remained a legal requirement in Czechia (until March 2012 under Act No. 20/1966 Coll. and Decree No. 19/1988 Coll., §10; since April 2012 under Act No. 372/2011 Coll.). In cases with suspected criminal aspects, autopsies were ordered by the police in accordance with §115 of the Code of Criminal Procedure (Act No. 141/1961 Coll.). For the included regions, autopsies were routinely performed at the regional institute in Olomouc; notably, there is no Institute of Forensic Medicine in Zlín.

We compiled yearly lists of cases involving pedestrian or cyclist fatalities in road traffic crashes. In earlier years, role identification was based on manual death registry logbooks; later, on ICD-coded entries. From each year, a random sample of 15 cases was selected to represent a full calendar year.

This randomisation reduces selection bias only within the archive-based sampling frame. It does not address possible selection bias in the archive itself, including temporal changes in which fatalities were submitted for autopsy, retained in the archive, or routed to the Olomouc institute. Consequently, random selection of 15 protocols per year should not be taken to mean that the sample fully represents all VRU fatalities in the two regions.

Key variables included sex, age, place and time of death (from the death certificate), role in the crash (from autopsy or police reports), and injury types and severity (classified by ISS and AIS). Crash characteristics—date, location, time, and crash type—were retrieved from police or accompanying documents.

Under Czech legislation (Act No. 372/2011, § 81(4a)), no consent from deceased individuals or their relatives is required for scientific research or teaching, provided that all data are anonymized and re‐identification is impossible. Our data do not allow for any re‐identification of deceased subjects.

The study protocol was reviewed and approved by the Ethics Committee of University Hospital Olomouc. The approval confirmation was provided to the journal editors.

Table 1 presents descriptive characteristics of our sample by time period. The sample consisted of 180 records while 118 (65.6%) of them concerned pedestrians and remaining 62 (34.4%) of them concerned cyclists. The distribution of pedestrians and cyclists remained stable. Males comprised nearly two thirds of all cases (64.4%). The mean age of deceased individuals was 49 years, with an increase from 44 in 1969–1984 to 56 years in 2009–2024. A shift was observed in place of death: deaths in a hospital decreased from 61.6% in 1969–1984 to 33.3% in 2009–2024, while deaths at the scene increased correspondingly.

**Table 1:** Descriptive characteristics of the study sample (deceased pedestrians and cyclists) by time period.

Characteristic	1969-1984 (n = 60)	1989-2004 (n = 60)	2009-2024 (n = 60)	Total (n = 180)
**Sex, n (%)**				
Female	20 (33.3%)	18 (30.0%)	26 (43.3%)	64 (35.6%)
Male	40 (66.7%)	42 (70.0%)	34 (56.7%)	116 (64.4%)
**Age, years**				
Mean (SD)	44 (26)	47 (23)	56 (21)	49 (24)
Median (Q1-Q3)	48 (19-70)	48 (29-68)	61 (42-74)	52 (28-71)
**Road user, n (%)**				
Pedestrian	42 (70.0%)	37 (61.7%)	39 (65.0%)	118 (65.6%)
Cyclist	18 (30.0%)	23 (38.3%)	21 (35.0%)	62 (34.4%)
**Place of death, n (%)**				
Scene	22 (36.7%)	31 (51.7%)	39 (65.0%)	92 (51.1%)
Hospital	37 (61.6%)	26 (43.3%)	20 (33.3%)	83 (46.1%)
Transport	1 (1.7%)	3 (5.0%)	1 (1.7%)	5 (2.8%)

Each record contained an information on severity of injuries in the form of the Injury Severity Score (ISS; [8]). Injury severity scoring (ISS) was reconstructed retrospectively from the written autopsy protocols. Across the study period, traumatic findings in these protocols are documented as a structured list of injuries, which provided a consistent basis for retrospective injury mapping. To minimise variability attributable to scoring practice, all protocols were reviewed and AIS-based injury severity was assigned by a single assessor using a standardised procedure, with subsequent ISS calculation performed using the same decision rules for cases from all decades. ISS is a standard medical measure of trauma severity (ranges from 3 to 75). It is defined as a sum of squares of three highest abbreviated injury scales (AIS), while each AIS ranges from 1 (minor severity) to 6 (maximal, currently untreatable, severity). If any of AIS equals 6, ISS is automatically set to 75.

**Fig 1:**
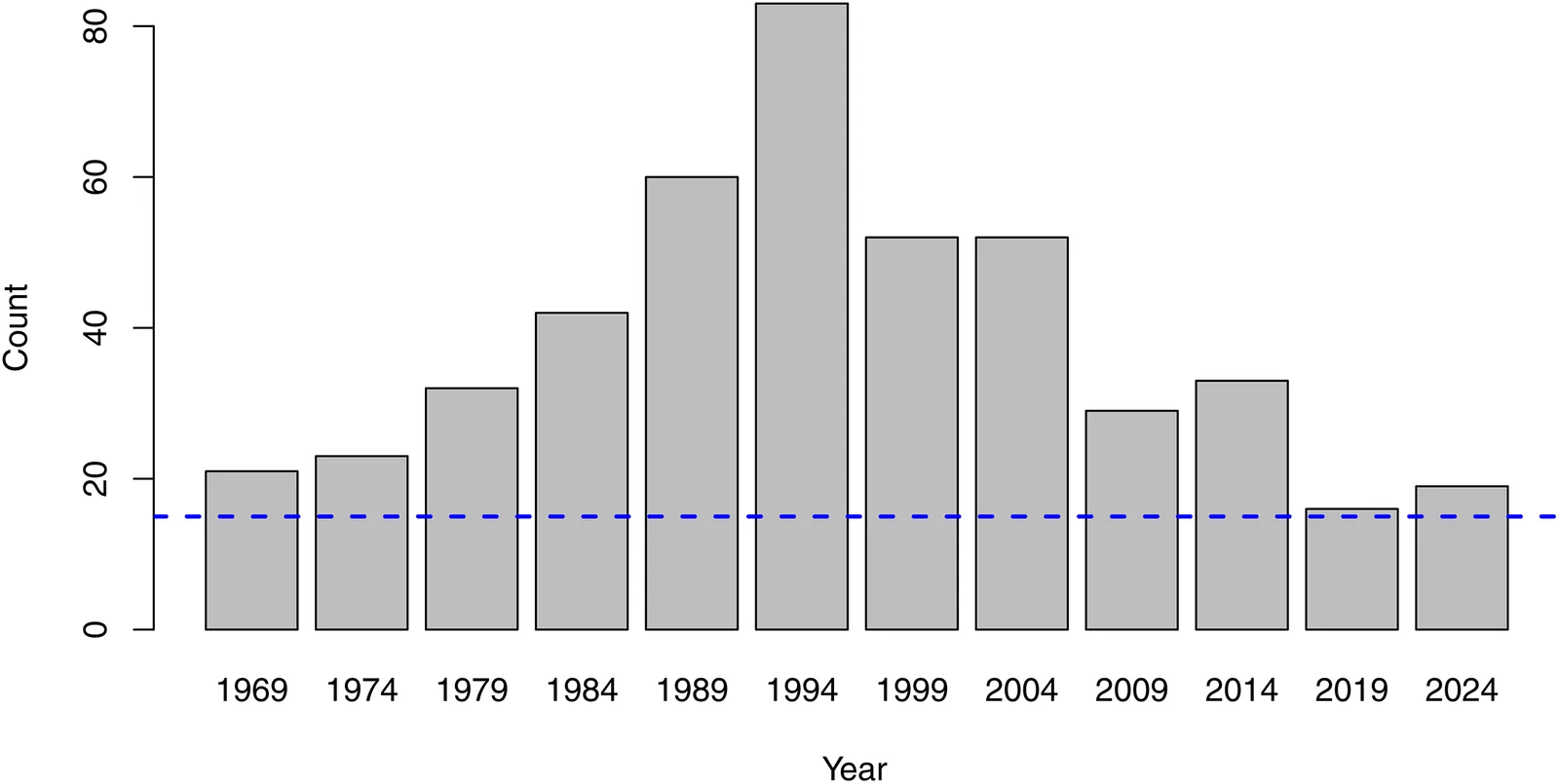
Total counts of records in every fifth year from period 1969 – 2024. In each considered year, 15 records were randomly selected.

Boxplots were used as a visualization tool for examining and comparing ISS distributions over the period in question. We were interested in time evolution of the first quartiles and minimum values of ISS. The first quartiles were estimated using the Harrell-Davis quantile estimator as it provides a robust estimate even for small samples [9]. The traditional minimum of ISS in the sample was used to estimate the minimum value of the population. While estimating the minimum value from a small sample is not reliable in general, the limitations become less critical when the population size is small as well. We used a fixed sample size (15 cases) per selected year to keep the effort of retrospective scoring feasible and to ensure that each year contributed equally to the long-term trend analysis rather than being dominated by years with higher case counts. In our case, a sample of 15 observations every fifth year (i.e., 180 observations in total) still represents almost two fifths (39%) of the population (see Fig 1), where “population” denotes the autopsied VRU fatalities in our sampling frame for the given years. Because the 15 protocols were selected at random from the yearly lists, inclusion probability within this frame was not influenced by place of death, crash type, or region.

The Mann-Kendall trend test [10,11] was applied to identify a consistent monotonic trend. The Mann-Kendall test is a non-parametric approach testing the null hypothesis H_0_: “No monotonic trend is present” against the alternative hypothesis H_A_: “A monotonic trend is present”. In general, the trend may be either linear or nonlinear. The Mann-Kendall test can be applied instead of the parametric linear regression which requires an assumption on the distribution of residuals from the fitted linear model.

If a monotonic trend is present in the data, we can approximate it by a linear trend and estimate its slope by a non-parametric method developed by Theil [12] and extended by Sen [13]. This Sen’s slope (or Theil-Sen estimator) naturally follows the Mann-Kendall test. The standard error of the Mann-Kendall test is used for calculating the confidence interval of the Sen’s slope. In the result, the Sen’s slope tells us the extent of increase/decrease per unit of time. In our case, it measures the increase/decrease of ISS per year.

A bootstrap [14] sensitivity analysis was performed to account for uncertainty due to small sample sizes and to assess the stability of the trend analysis. We utilized bootstrap approach (10,000 iterations) stratified by year (i.e., resampling records within each year separately) to preserve the temporal structure of the data. This process generated bootstrap distributions for both the p-values of the Mann-Kendall trend test and the Sen’s slope estimates. From these distributions, bootstrap medians and bootstrap 95% confidence intervals were derived.

R software [15] was used for all computations along with packages “Hmisc” [16] for the Harrell-Davis quantile estimator, “Kendall” [17], performing the Mann-Kendall test, “mblm” [18], building median-based linear models and calculating Sen’s slopes, and “boot” [19], for the bootstrap sensitivity analysis.

## Results

**Fig 2:**
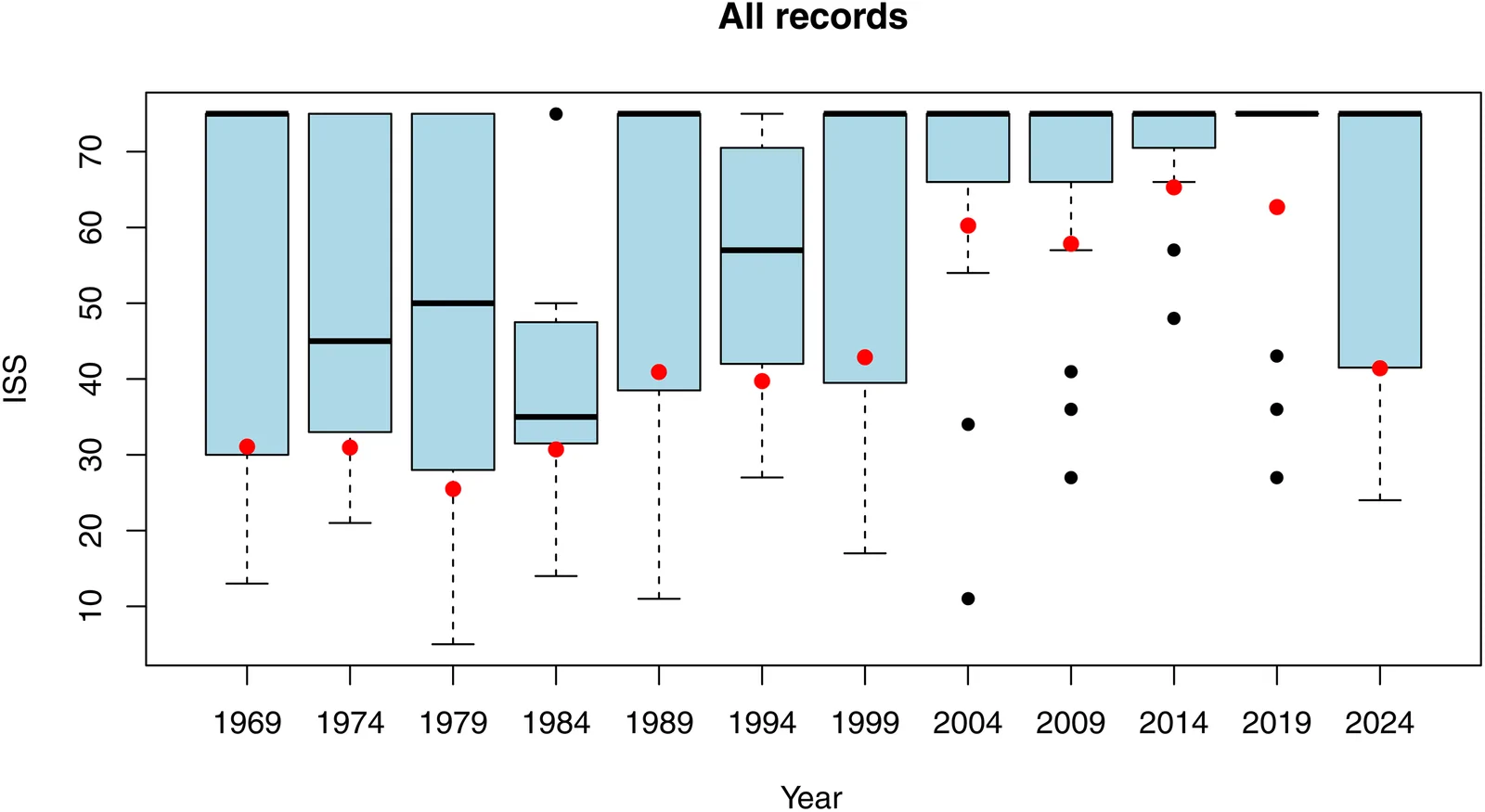
Boxplots of ISS for all records across the period 1969 – 2024 along with the Harrell-Davis estimate of the first quartile (red points).

**Fig 3:**
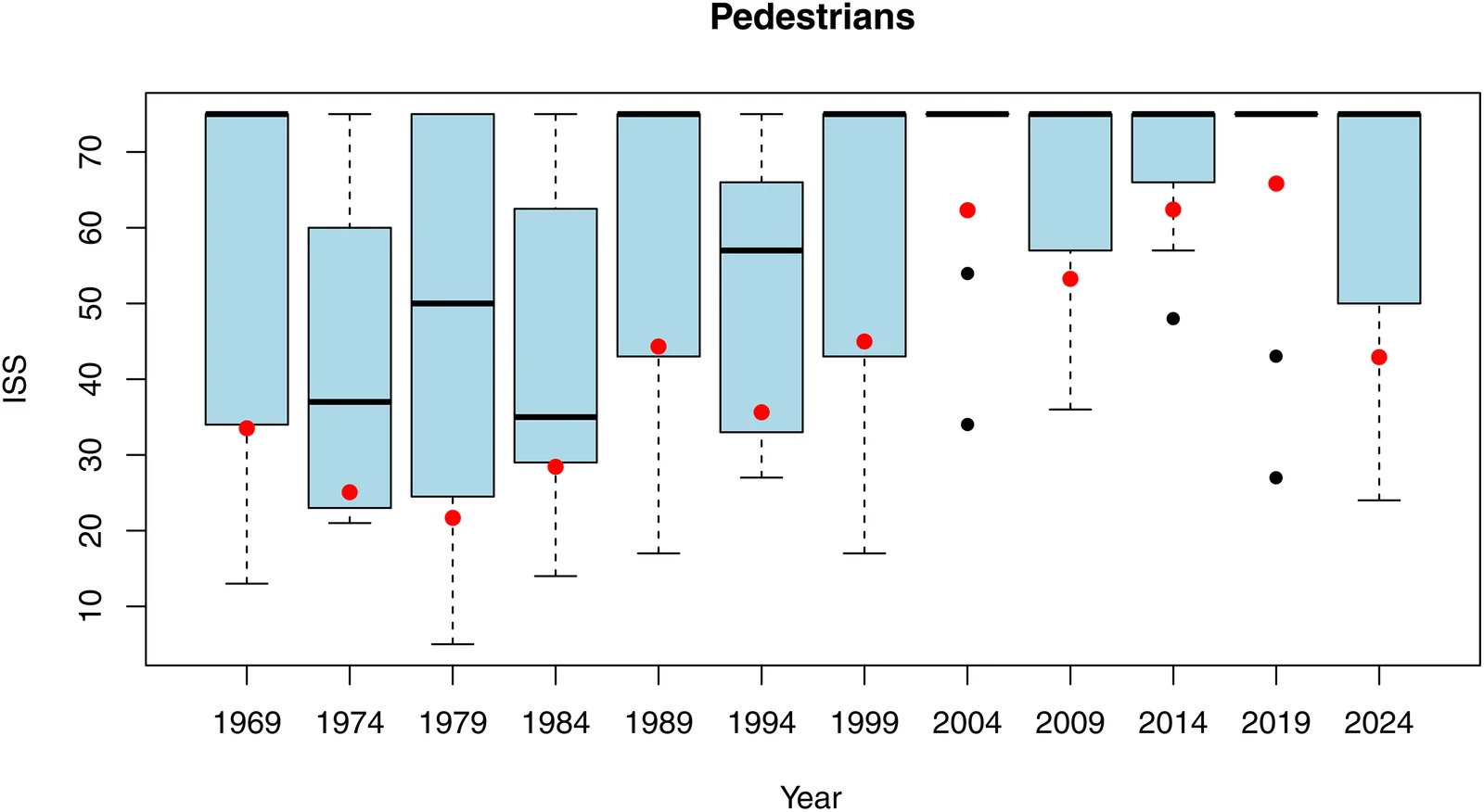
Boxplots of ISS for pedestrians across the period 1969 – 2024 along with the Harrell-Davis estimate of the first quartile (red points).

**Fig 4:**
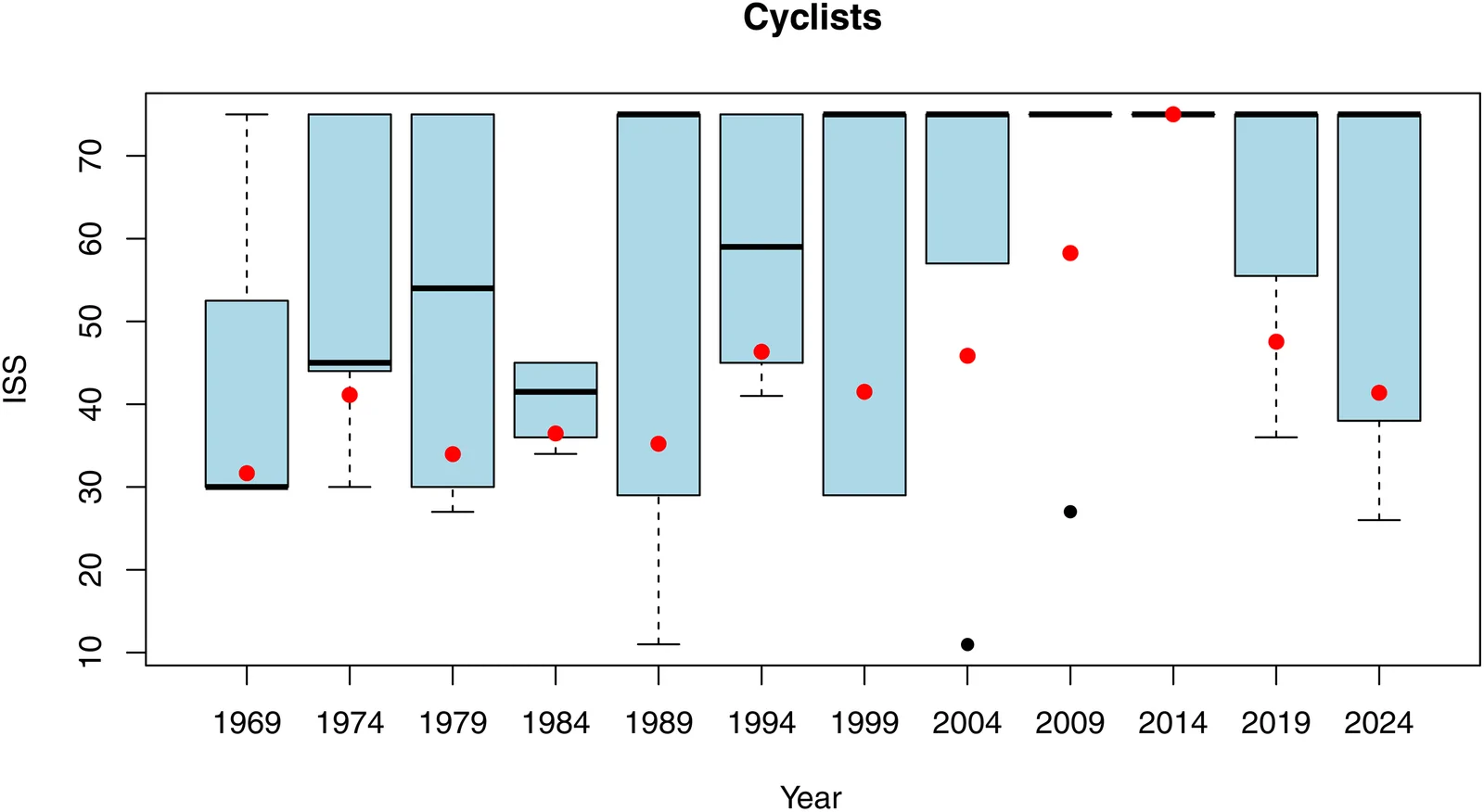
Boxplots of ISS for cyclists across the period 1969 – 2024 along with the Harrell-Davis estimate of the first quartile (red points).

The majority (56.1%) of records has ISS of the maximum possible value. Only in 4 out of 12 years, the median of ISS was lower than 75 (Fig 2). Hence, it does not make sense to analyse the time evolution of medians. The same remains true also for separate groups of both pedestrians (Fig 3) and cyclists (Fig 4). Therefore, we focused on the trend identification and estimation only for the first quartile, depicted as red points (Harrell-Davis estimate), and for the minimum values of ISS.

Considering all records, a monotonic trend for the first quartile was identified. Concerning pedestrians, a monotonic trend was found for both the first quartile and minimum values of ISS. For cyclists, the original Mann-Kendall test returned a statistically significant result for the first quartile. However, bootstrap sensitivity analysis revealed substantial uncertainty (see Table 2 for all resulting p-values of the Mann-Kendall test).

**Table 2:** Results of the Mann-Kendall trend test with bootstrap validation for minimum ISS (Min) and the first quartile ISS (Q25). Bootstrap estimates based on 10,000 iterations with stratified resampling. Statistical significance determined using Bonferroni-corrected threshold within each family of three tests (α = 0.05/3 = 0.0167). *Statistically significant result according to the original p-value but bootstrap 95% CI indicates uncertainty.

Group	Metric	p-value (original)	p-value (bootstrap median)	Bootstrap 95% CI for p-value	Significant
All records	Min	0.0830	0.0850	[0.0108, 0.6273]	No
	Q25	0.0075	0.0335	[0.0013, 0.3727]	Yes*
Pedestrians	Min	0.0159	0.0230	[0.0006, 0.3336]	Yes*
	Q25	0.0049	0.0335	[0.0008, 0.4507]	Yes*
Cyclists	Min	1.0000	0.6786	[0.1226, 1.0000]	No
	Q25	0.0112	0.1499	[0.0060, 0.9442]	Yes*

The original Mann-Kendall test suggested four statistically significant trends (Table 2); however, bootstrap sensitivity analysis indicated that statistical support was uncertain, particularly for cyclists Q25. Despite this uncertainty, three of the four cases demonstrated robust increasing trends across all bootstrap samples (Table 3). In the remaining case (cyclists Q25), the bootstrap confidence interval for Sen’s slope included zero (95% CI: [−0.22, 0.78]), indicating uncertainty in the trend direction.

**Table 3:** Sen’s slope estimates with bootstrap validation for metrics showing evidence of a temporal trend (Table 2). Bootstrap estimates based on 10,000 iterations.

Group	Metric	Slope (original)	Slope (bootstrap median)	Bootstrap 95% CI for slope	Interpretation
All records	Q25	0.83	0.70	[0.53, 1.27]	Increasing
Pedestrians	Min	0.60	0.60	[0.17, 0.84]	Increasing
	Q25	0.92	0.77	[0.65, 1.43]	Increasing
Cyclists	Q25	0.36	0.43	[-0.22, 0.78]	No trend

Concerning the age of the pedestrians and cyclists, no statistically significant trend in the median values was observed (Mann-Kendall trend test, p-value = 0.5800). It suggests that the medians fluctuated without any consistent monotonic change. These variations were random rather than part of a long-term pattern.

## Discussion

This study set out to track five-decade trends in fatal injury severity among VRUs. Our findings reveal a long-term upward trend in the lower range of injury severity (first quartile ISS) among deceased VRUs in two Czech regions, while evidence for a monotonic trend in minimum ISS was limited to pedestrians. A survivorship or fatality-composition mechanism — in which individuals with less critical injuries increasingly survive — is one plausible explanation, but the data do not allow us to determine whether this was driven primarily by post-crash care. The observed trend should therefore be interpreted as an association compatible with improved survivability, while recognising that changes in crash severity, exposure, case mix, referral pathways, reporting, and documentation may also have contributed. Consistent with our data, similar shifts have been documented in high-income countries, where the development of organised trauma systems, faster EMS response times, and improved prehospital care have led to declines in preventable trauma-related deaths [6,20]. For instance, studies in the United States and Germany have shown that over time, trauma fatalities tend to involve higher ISS values, reflecting better survival odds for less severely injured patients [21].

These comparisons provide contextual motivation only. Our study contributes an archive-based long-term case series from two Czech regions to a broader regional evidence gap, but it does not support population-level inference for Central Europe or Central and Eastern Europe.

Vulnerable road users (VRUs) are particularly sensitive to changes in the quality and efficiency of emergency care, as they lack the structural protection afforded to motor vehicle occupants. Prior studies have shown that VRUs not only face higher risks of death and serious injury but also rely more heavily on timely and effective post-crash response systems [1,2,5]. The upward shift in Q25 ISS among fatal VRU cases is best described as consistent with a possible survivorship/composition shift rather than as evidence that moderately severe injuries are now more likely to survive. Likewise, the change in place of death should be treated as a descriptive contextual finding only. Because place of death can be influenced by crash severity, transport decisions, referral practices, hospital admission patterns, reporting rules, and case documentation, it should not be presented as triangulating evidence that post-crash care improved.

Several mechanisms other than post-crash survival improvements could also influence long-term severity patterns among fatalities. These include changes in the underlying crash mix (e.g., impact speeds, urban/rural exposure, vehicle fleet composition), demographic shifts among VRUs (e.g., ageing), and potential changes in documentation practices across decades. In our setting, the stable catchment of a single institution helps limit geographic discontinuities, but we cannot fully disentangle exposure changes from medical-system effects. We therefore interpret increasing Q25 ISS primarily as evidence consistent with improved survivability, while acknowledging that concurrent shifts in crash composition may contribute to the observed pattern. However, a stable pedestrian/cyclist distribution and no statistically significant monotonic trend in median age in our sample make simple compositional ageing or VRU-mix changes less likely as the primary explanation. Beyond these factors, VRU outcomes can also shift over time due to changes in the speed environment and impact circumstances (e.g., speed management and enforcement), vehicle fleet composition (e.g., heavier vehicles), road and junction design, alcohol prevalence, and exposure patterns (e.g., cycling uptake and route choice). These mechanisms could bias our inference in either direction: if crashes became more severe (higher speeds, heavier vehicles), they could increase Q25 ISS among fatalities independently of post-crash care (biasing the survivability inference upward); conversely, if the crash environment became less severe (speed calming, safer infrastructure), it could dampen or mask the extent to which post-crash survival improved (biasing the survivability inference downward).

### Study limitations

Our dataset is restricted to VRU fatalities that underwent autopsy within the institutional archive used for sampling. Therefore, the results should be generalised to all VRU fatalities in the two regions with caution, particularly if the proportion of fatalities undergoing autopsy (or being routed to this institute) varied over time. While Czech law has long required autopsies for sudden, violent, or suspicious deaths, we cannot fully exclude temporal changes in autopsy indications or administrative practices that could influence representativeness. Given the largely national nature of EMS and trauma-system development in Czechia, the observed pattern may be relevant to broader national discussions, but it should not be interpreted as direct evidence of national trends.

In addition, random sampling within the archive cannot eliminate potential selection bias arising before cases entered the archive. Over a five-decade period, the set of fatalities recorded in a single institutional archive may have been influenced by changes in autopsy practices, legal or administrative referral criteria, institutional catchment, inter-regional case routing, record preservation, or documentation systems. These factors could affect the composition of archived fatalities independently of true changes in VRU fatal injury patterns. Therefore, the findings should be interpreted as trends within an archive-based autopsied fatality series rather than as fully representative estimates for all VRU fatalities in the two regions.

A further limitation is that ISS was derived retrospectively from archival documentation spanning 1969–2024. Although the autopsy protocols used for scoring showed a stable structure over time and injury severity coding was applied using a single standardised approach, changes over five decades in clinical diagnostics, documentation practices, and the evolution of AIS/ISS conventions could still introduce residual measurement heterogeneity. Consequently, apparent long-term trends in ISS should be interpreted cautiously, as a proportion of the observed change may reflect differences in injury ascertainment and recording rather than solely true changes in injury severity.

Spatial analysis was not feasible due to lack of geocoded incident data in older records. Information on individuals who died as a result of traffic accidents was available solely through medical records, which, particularly in older cases (prior to 2020), often lacked precise details regarding the location of the injury. As a result, it was not possible to geographically locate the incidents or assign them to specific spatial units. This significantly limited the ability to conduct spatial analysis or account for environmental characteristics.

The study by [22] demonstrated that spatially detailed data (at the ward level) enable the examination of relationships between area characteristics—such as population density, land use types, levels of social deprivation, and the road network—and the likelihood of traffic accidents. However, in our case, comparable spatial data were not available, not even in approximate forms such as accident locations, patient residences, or ambulance dispatch sites.

Although all data originated from a single hospital with a relatively stable catchment area, this alone was insufficient to support meaningful spatial analysis of environmental influences. For these reasons, it was not possible to replicate the methodological approach used in the aforementioned study.

Any control group, e.g., of severely injured individuals, was not included in the analysis because data on non-fatal road traffic accidents (i.e., survivors with severe injuries) have been systematically available in the Czech Republic only since 2006. In contrast, data on fatalities are available for the entire study period from 1969 onwards. For this reason, it was not possible to create a comparable control group for the full time series, which represents a methodological limitation of this study.

### Implications for Czechia and comparable contexts

Although our severity-based proxy cannot isolate individual interventions, the observed upward shift in Q25 ISS among VRU fatalities is most consistent with improvements across the rescue chain, particularly (i) timely dispatch and response-time coverage, (ii) structured prehospital triage and on-scene stabilisation (including physician-led support where available), and (iii) rapid access to definitive emergency and trauma care through regionalised hospital pathways. For Czechia, these findings support continued emphasis on system-wide performance (coverage, coordination, and training) rather than single-point interventions.

When applying this proxy approach elsewhere, the first step should be to document not only the random sampling procedure but also the stability and completeness of the archive itself. This includes describing catchment changes, autopsy referral rules, institutional responsibilities, record retention, and any periods in which coverage may have differed. Without this information, an apparently random archive sample may still be affected by upstream selection processes.

- **Minimum data standards** for applying this approach include: year (or date) of crash/death, VRU role (pedestrian/cyclist), age and sex, a structured injury description sufficient for AIS/ISS mapping, and place of death (scene/transport/hospital).
- **Recommended enhancements** to strengthen interpretability include: geocoding (or at least municipality/road class), crash mechanism/type, toxicology/alcohol indicators, and prehospital time stamps (call receipt, dispatch, arrival, departure, hospital arrival) enabling direct linkage to EMS performance indicators.

Directly building on the limitations of this study, future Czech surveillance and research would benefit from (i) routine capture of geocoded crash location (or standardised approximate location fields) in medical/forensic documentation to enable spatial analyses of infrastructure and access-to-care, and (ii) linkage of fatal and non-fatal serious-injury records (available systematically since 2006) across police, EMS dispatch, hospital/trauma registries, and forensic datasets. Such linkage would allow parallel trend analyses for severely injured survivors versus fatalities, providing a stronger test of the post-crash survivability hypothesis and helping separate medical-system effects from changes in crash exposure and environment.

With only twelve time points combined with low sample sizes in each year, the trend tests had only limited statistical power. However, consistent slope estimates across all bootstrap samples provided robust evidence of increasing ISS trends for Q25 in the full sample of records and for pedestrians, whereas evidence for a minimum-ISS trend was observed only for pedestrians.

In summary, over the past five decades fatally injured VRUs have exhibited significantly higher first-quartile ISS values, indicating an upward shift in the lower tail of injury severity among fatal cases. This increase in Q25 was observed in the full set of records and when analysing pedestrians separately; for cyclists, the Q25 trend showed greater uncertainty in bootstrap validation. A statistically significant increasing trend in minimum ISS was found only for pedestrians, with no corresponding minimum-ISS trend for all records or for cyclists. This shift cannot be attributed to changes in victim age, which remained statistically stable throughout the study period. The pattern is consistent with a survivorship/composition shift in which individuals with less critical injuries increasingly survive, but it does not by itself demonstrate that post-crash care was the primary driver — alternative explanations related to crash severity, exposure, case mix, referral and reporting practices, autopsy selection, and documentation remain plausible. Place of death should therefore be reported only as a descriptive characteristic of the sample, not as confirmatory evidence of improved post-crash survivability. Future research should incorporate non-fatal serious injuries and linked EMS, hospital, police, and forensic data to test the survivability hypothesis more directly.

## Supporting information

S1 Data Support data ISS-en light. (XLSX)
